# Supplementary material for: Sulfonamidoboronic Acids as “Cross-Class” Inhibitors of an Expanded-Spectrum Class C Cephalosporinase, ADC-33, and a Class D Carbapenemase, OXA-24/40: Strategic Compound Design to Combat Resistance in Acinetobacter baumannii
Source: Antibiotics (Basel). 2023 Mar 24;12(4):644. doi: 10.3390/antibiotics12040644 (PMC10135033; doi:10.3390/antibiotics12040644)

Sulfonamidoboronic Acids as Cross-Class Inhibitors of ADC-33 and OXA-24/40 ß-lactamases to Combat Resistance in *Acinetobacter baumannii*

Maria Luisa Introvigne, Trevor J. Beardsley, Micah C. Fernando, David A. Leonard, Bradley J. Wallar, Susan D. Rudin, Magdalena A. Taracila, Philip N. Rather, Jennifer M. Colquhoun, Shaina Song, Francesco Fini, Kristine M. Hujer, Andrea M. Hujer, Fabio Prati, Rachel A. Powers, Robert A. Bonomo, Emilia Caselli

Corresponding authors: emilia.caselli@unimore.it

Supporting Information

Contents

Synthesis of compounds 1a-c, **2a-c**, **3a-c**, **5a-e** and **6a-e** S2-S6

Supplemental Figure S1. Walleye stereoviews of the Polder omit maps for OXA-24/40 and ADC-33 in complex with **CR167**, **6d** and **6e** S7-S9

Synthesis of pinanediol alkyl boronates **1a-c**. (+)- or (–)- Pinanediol (16.7 mmol, 1 eq.) was added to a solution of alkylboronic acids (16.7 mmol, 1 eq.) in dry THF (15 mL). The mixture was stirred at room temperature for 5h. The THF was evaporated in vacuo. The crude was re-solubilized in CH_2Cl_2 and Na_2SO_4 was added. The solution was dried over Na_2SO_4 for 20-30 min then it was filtered and evaporated. The crude product was purified by column chromatography (petroleum ether /diethyl ether 95:5).

(–)-Pinanediolmethaneboronate (**1a**). Colourless oil (68%). ^{1}H NMR (400 MHz, $CDCl_3$) δ: 4.25 (dd, J = 8.7, 1.8 Hz, 1H, C*H*OB), 2.39 – 2.28 (m, 1H, $CH_{2\text{-pin}}$), 2.27 – 2.16 (m, 1H, $CH_{2\text{-pin}}$), 2.04 (t, J = 5.5 Hz, 1H, CH_{pin}), 1.91 (dt, J = 8.6, 4.5 Hz, 1H, CH_{pin}), 1.87 – 1.81 (m, 1H, $CH_{2\text{-pin}}$), 1.54 (s, 3H, CH_3), 1.39 (s, 3H, $CH_{3\text{-pin}}$), 1.29 (s, 3H, $CH_{3\text{-pin}}$), 1.13 (d, J = 10.9 Hz, 1H, $CH_{2\text{-pin}}$), 0.84 (s, 3H, $CH_{3\text{-pin}}$).^{13}C NMR (101 MHz, $CDCl_3$) δ: 85.56, 77.81, 51.49, 39.70, 38.29, 35.63, 28.83, 27.25, 26.60, 24.14. ^{11}B NMR (400 MHz, $CDCl_3$) δ: 33.42.

(–)-Pinanediolethaneboronate (**1b**). Colourless oil (54%). ^{1}H NMR (400 MHz, $CDCl_3$) δ: 4.25 (dd, J = 8.7, 1.9 Hz, 1H, CH_{pin}), 2.39 – 2.28 (m, 1H, $CH_{2\text{-pin}}$), 2.21 (m, 1H, $CH_{2\text{-pin}}$), 2.04 (t, J = 5.6 Hz, 1H, CH_{pin}), 1.91 (dt, J = 5.6, 2.9 Hz, 1H, CH_{pin}), 1.88 – 1.79 (m, 1H, $CH_{2\text{-pin}}$), 1.38 (s, 3H, $CH_{3\text{-pin}}$), 1.29 (s, 3H, $CH_{3\text{-pin}}$), 1.11 (d, J = 10.9 Hz, 1H, $CH_{2\text{-pin}}$), 0.97 (t, J = 7.8 Hz, 3H, CH_3), 0.84 (s, 3H, $CH_{3\text{-pin}}$), 0.82 – 0.74 (m, 2H, CH_2B).^{13}C NMR (101 MHz, $CDCl_3$) δ: 85.46, 77.74, 51.48, 39.69, 38.28, 35.70, 28.83, 27.24, 26.57, 24.14, 8.01. ^{11}B NMR (400 MHz, $CDCl_3$) δ: 33.94.

(+)-Pinanediolethaneboronate (**1c**). Colourless oil (91%). Spectroscopic properties were identical to **1b**.

Synthesis of α–chloroalkylboronates **2a-c**. Freshly distilled CH_2Cl_2 (8.24 mmol, 2 eq.) and dry THF (11 mL) were added in a four-necked flask under argon. The temperature was cooled to -100 °C using a 1:1 EtOH/MeOH bath and liquid N_2. n-Butyllithium (4.94 mmol, 1.2 eq.) was added dropwise and the mixture was stirred at -100 °C. After 20 min the selected pinanediol alkylboronate 1 (4.12 mmol, 1 eq.) was dissolved in dry THF and added dropwise to the mixture. The temperature was allowed to reach -80 °C and $ZnCl_2$ (3.3 mmol, 0.8 eq.) was added dropwise. The temperature was allowed to warm overnight, then the EtOH/MeOH bath was removed and the mixture was stirred for 1h at room temperature. The mixture was partitioned between saturated NH_4Cl (20 mL) and petroleum ether (70 mL). The organic phase was washed twice with saturated NH_4Cl (2 x 20 mL), then dried over Na_2SO_4, filtered and evaporated in vacuo. The crude product was not purified and used directly in the next reaction.

General procedure for the synthesis of silylamines **3a-c**. The selected α-chloroboronate **2** (1.47 mmol, 1 eq.) was dissolved in dry THF (4.40 mL) under Ar. The solution was cooled to -100 °C using a 1:1 EtOH/MeOH bath and liquid N_2. Lithium bis-trimethylsilylamide (1.61 mmol, 1.1 eq.) was added dropwise. The temperature was allowed to warm overnight, then the bath was removed and the mixture was stirred at room temperature for 1h. The mixture was partitioned between H_2O (4 mL) and petroleum ether (15 mL). The

aqueous phase was re-extracted twice with petroleum ether (2 x 10 mL), then the organic phases were washed with saturated NaCl, dried over Na_2SO_4, filtered, and evaporated in vacuo.

(–)-Pinanediol (1*S*)-1-(N-bis(trimethylsilyl)amino)-ethaneboronate (**3a**). Yellow oil (56%). ^{1}H NMR (400 MHz, $CDCl_3$) δ: 4.33 (d, *J* = 3.6 Hz, CH_{pin}), 2.39-1.86 (m, 5H, pinanediol), 1.42 (s, 3H, $CH_{3\text{-}pin}$), 1.31 (s, 3H, $CH_{3\text{-}pin}$), 1.22 (d, *J* = 7.5 Hz, 3H, CH_3), 1.17 (d, *J* = 10.9 Hz, 1H, $CH_{2\text{-}pin}$), 0.87 (s, 3H, $CH_{3\text{-}pin}$), 0.13 (s, 18H, TMS_2). ^{13}C NMR (101 MHz, $CDCl_3$) δ: 85.68, 78.32, 51.47, 39.50, 38.20, 37.00 (CB), 35.45, 28.41, 27.08, 26.28, 24.02, 21.28, 2.67.

(–)-Pinanediol (1*S*)-1-(N-bis(trimethylsilyl)amino)-propaneboronate (**3b**). Yellow oil (85%). ^{1}H NMR (400 MHz, $CDCl_3$) δ: 4.23 (dd, *J* = 8.6, 1.8 Hz, 1H, CH_{pin}), 2.71 (t, *J* = 7.1 Hz, 1H, CHB), 2.36 – 2.27 (m, 1H, $CH_{2\text{-}pin}$), 2.26 – 2.15 (m, 1H, $CH_{2\text{-}pin}$), 2.02 (dd, *J* = 10.3, 5.1 Hz, 1H, CH_{pin}), 1.90 (dd, *J* = 5.6, 2.6 Hz, 1H, CH_{pin}), 1.87 – 1.78 (m, 1H, $CH_{2\text{-}pin}$), 1.52 (dd, *J* = 14.5, 7.2 Hz, 2H, CH_2), 1.38 (s, 3H, $CH_{3\text{-}pin}$), 1.32 (s, 3H, $CH_{3\text{-}pin}$), 1.22 (d, *J* = 10.9 Hz, 1H, $CH_{2\text{-}pin}$), 0.93 (td, *J* = 7.4, 2.2 Hz, 3H, CH_3), 0.83 (s, 3H, $CH_{3\text{-}pin}$), 0.18 (s, 9H, TMS), 0.15 (s, 9H, TMS). ^{13}C NMR (101 MHz, $CDCl_3$) δ: 83.71, 78.34, 51.60, 44.35, 40.08, 38.50, 35.58, 28.75, 28.57, 28.49, 27.28, 26.56, 24.15, 12.68, 1.49, 1.01.

(+)-Pinanediol (1*R*)-1-(N-bis(trimethylsilyl)amino)-propaneboronate (**3c**). Yellow oil (81%). Spectroscopic properties were identical to **3b**.

General procedure for the synthesis of hydrochlorides (**4a-c**). The selected bis-trimethylsilylamide **3** (0.69 mmol, 1 eq.) was dissolved in dry THF (4.1 mL) under Ar. The temperature was cooled to 0 °C with an ice bath and HCl 4 M in dioxane (0.6 mL) was slowly added. The reaction was allowed to reach room temperature and stirred under Ar for 4h. The mixture was evaporated to dryness in vacuo. The crude product **4** was purified by trituration with dry Et_2O.

(–)-Pinanediol (1*S*)-1-aminoethaneboronate hydrochloride (**4a**). White solid (100%). ^{1}H NMR (400 MHz, DMSO-d_6) δ: 7.82 (s, 3H, NH_3), 4.46 (dd, *J* = 8.7, 1.8 Hz, 1H, CH_{pin}), 2.87 (dd, *J* = 7.4, 5.9 Hz, 1H, CHB), 2.39 – 2.26 (m, 1H, $CH_{2\text{-}pin}$), 2.20 (ddd, *J* = 10.8, 6.2, 2.0 Hz, 1H, $CH_{2\text{-}pin}$), 2.01 (t, *J* = 5.5 Hz, 1H, CH_{pin}), 1.91 – 1.81 (m, 1H, CH_{pin}), 1.81 – 1.73 (m, 1H, $CH_{2\text{-}pin}$), 1.38 (s, 3H, $CH_{3\text{-}pin}$), 1.30 (s, 3H, $CH_{3\text{-}pin}$), 1.23 (d, *J* = 7.7 Hz, 3H, CH_3), 1.17 – 1.10 (m, 1H, $CH_{2\text{-}pin}$), 0.86 (s, 3H, $CH_{3\text{-}pin}$). ^{13}C NMR (101 MHz, DMSO-d_6) δ: 86.72, 77.67, 51.21, 50.70, 37.90, 35.54, 34.59 (CB), 28.45, 26.88, 25.89, 23.62, 14.53.

(–)-Pinanediol (1*S*)-1-aminopropaneboronate hydrochloride (**4b**). White solid (91%). ^{1}H NMR (400 MHz, DMSO-d_6) δ: 7.91 (s, 3H, NH_3); 4.45 (dd, *J* = 8.8, 2.0 Hz, 1H, CH_{pin}); 2.72 (m, 1H, CHB); 2.30 - 1.77 (m, 5H, pinanediol), 1.67 (p, *J* = 7.4 Hz, 2H, CH_2); 1.38 (s, 3H, $CH_{3\text{-}pin}$); 1.27 (s, 3H, $CH_{3\text{-}pin}$); 1.14 (d, *J* = 10.8 Hz, 1H, $CH_{2\text{-}pin}$); 0.95 (t, *J* = 7.5 Hz, 3H, CH_3); 0.83 (s, 3H, $CH_{3\text{-}pin}$). ^{13}C NMR (101 MHz, DMSO-d_6) δ: 86.74, 77.56, 50.67, 38.93, 38.82, 37.83 (CB), 34.65, 28.21, 26.80, 25.92, 23.59, 22.46, 10.93. ^{11}B NMR (400 MHz, DMSO-d_6) δ: 33.71.

(+)-Pinanediol (1*R*)-1-aminopropaneboronate hydrochloride (**4c**). White solid (100%). Spectroscopic properties were identical to **4b**.

General procedure for the synthesis of pinanendiol sulfonamidoboronates (**5**). The selected sulphonyl chloride (1.46 mmol, 2 eq.) was added to a solution of the desired hydrochloride **4** (0.73 mmol, 1 eq.) in dry CH_2Cl_2 (11 mL) under Ar. DIPEA (1.60 mmol, 2.2 eq.) was solubilized in dry CH_2Cl_2 (2 mL) and added dropwise to the mixture. The reaction was stirred at room temperature for 4h. CH_2Cl_2 (15 mL) and water (5 mL) were added to the mixture. The two phases were separated and the aqueous phase was re-extracted with CH_2Cl_2 (2 x 10 mL). The organic phases were washed with saturated NaCl, dried over Na_2SO_4, filtered and evaporated under reduced pressure. The crude was purified by column chromatography on silica gel (petroleum ether / diethyl ether 7:3), affording the desired product.

(–)-Pinanediol (1*S*)-1-(phenylmethanesulfonylamino)-ethaneboronate (**5a**). Yellowish solid (56%). ^{1}H NMR (400 MHz, $CDCl_3$) δ: 7.45 – 7.31 (m, 5H, CH_{phen}), 4.29 (d + t, *J* = 23.1, 11.3 Hz, 3H, CH_{pin} + CH_2SO_2), 3.16 – 3.02 (m, 1H, CHB), 2.38 – 2.30 (m, 1H, $CH_{2\text{-}pin}$), 2.29 – 2.20 (m, 1H, $CH_{2\text{-}pin}$), 2.04 (q, *J* = 5.1 Hz, 1H, CH_{pin}), 1.92 (d, *J* = 12.3 Hz, 1H, CH_{pin}), 1.84 (d, *J* = 14.8 Hz, 1H, $CH_{2\text{-}pin}$), 1.41 (s, 3H, $CH_{3\text{-}pin}$), 1.29 (d, *J* = 7.6, 4.7 Hz + s, 6H, CH_3 + $CH_{3\text{-}pin}$), 1.09 (d, *J* = 11.0 Hz, 1H, $CH_{2\text{-}pin}$), 0.84 (d, *J* = 4.2 Hz, 3H, $CH_{3\text{-}pin}$).^{13}C NMR (101 MHz, $CDCl_3$) δ: 130.91, 129.90, 128.80, 128.68, 87.21, 78.80, 59.55, 51.30, 39.53, 38.34, 35.85 (CB), 35.35, 28.63, 27.15, 26.46, 24.09, 18.89. ^{11}B NMR (400 MHz, $CDCl_3$) δ: 32.67. $[\alpha]^{20}_D$ = - 3.6 (MeOH).

(–)-Pinanediol (1*S*)-1-[3-(methoxycarbonyl)phenylmethanesulfonylamino]-ethaneboronate (**5b**). Yellowish solid (52%). ^{1}H NMR (400 MHz, $CDCl_3$) δ: 8.08 (s, 1H, CH_{phen}), 8.04 (d, *J* = 7.8 Hz, 1H, CH_{phen}), 7.64 (d, *J* = 7.5 Hz, 1H, CH_{phen}), 7.45 (t, *J* = 7.7 Hz, 1H, CH_{phen}), 4.32 (td, *J* = 14.0, 6.3 Hz, 3H, CH_{pin} + CH_2SO_2), 3.92 (s, 3H, OCH_3), 3.15 (dd, *J* = 13.3, 7.1 Hz, 1H, CHB), 2.34 (dd, *J* = 14.6, 8.9 Hz, 1H, $CH_{2\text{-}pin}$), 2.25 (dd, *J* = 11.4, 6.8 Hz, 1H, $CH_{2\text{-}pin}$), 2.04 (d, *J* = 6.6 Hz, 1H, CH_{pin}), 1.95-1.90 (m, 1H, CH_{pin}), 1.83 (d, *J* = 14.8 Hz, 1H, $CH_{2\text{-}pin}$), 1.41 (s, 3H, $CH_{3\text{-}pin}$), 1.30 (t, *J* = 7.5 Hz, 6H, $CH_{3\text{-}pin}$ + CH_3), 1.07 (d, *J* = 11.0 Hz, 1H, $CH_{2\text{-}pin}$), 0.84 (s, 3H, $CH_{3\text{-}pin}$). ^{13}C NMR (101 MHz, $CDCl_3$) δ: 166.68, 135.33, 132.05, 130.82, 130.40, 129.90, 128.90, 87.29, 78.80, 59.31, 52.37, 51.29, 39.53 (CB), 38.33, 35.34, 28.57, 27.14, 26.49, 24.09, 18.78. ^{11}B NMR (400 MHz, $CDCl_3$) δ: 32.92. $[\alpha]^{20}_D$ = -3.9 (MeOH)

(–)-Pinanediol (1*S*)-1-(phenylmethanesulfonylamino)-propaneboronate (**5c**). Yellowish solid (76%). ^{1}H NMR (400 MHz, $CDCl_3$) δ: 7.41 (dq, *J* = 6.7, 4.1, 3.3 Hz, 2H, CH_{phen}), 7.38 – 7.33 (m, 3H, CH_{phen}), 4.37 (dd, *J* = 8.8, 2.1 Hz, 1H, CH_{pin}), 4.32 (d, *J* = 3.3 Hz, 2H, CH_2SO_2), 4.27 (d, *J* = 6.4 Hz, 1H, NH), 3.05 (q, *J* = 6.0 Hz, 1H, CHB), 2.35 (ddt, *J* = 14.4, 8.9, 2.6 Hz, 1H, $CH_{2\text{-}pin}$), 2.25 (m, 1H, $CH_{2\text{-}pin}$), 2.06 (t, *J* = 5.5 Hz, 1H, CH_{pin}), 1.94 (m, 1H, CH_{pin}), 1.84 (dt, *J* = 14.9, 2.6 Hz, 1H, $CH_{2\text{-}pin}$), 1.74 (dt, *J* = 14.1, 7.1 Hz, 2H, CH_2), 1.41 (s, 3H, $CH_{3\text{-}pin}$), 1.30 (s, 3H, $CH_{3\text{-}pin}$), 1.14 (d, *J* = 10.9 Hz, 1H, $CH_{2\text{-}pin}$), 0.92 (t, *J* = 7.4 Hz, 3H, CH_3), 0.85 (s, 3H, $CH_{3\text{-}pin}$). ^{13}C NMR (101 MHz, $CDCl_3$) δ:130.89, 129.88, 128.78, 128.66, 87.19, 78.71, 59.31,

51.27, 39.57, 38.37, 35.42, 28.80, 27.17, 26.62, 25.95, 24.14, 10.89. ^{11}B NMR (400 MHz, $CDCl_3$) δ: 32.59. $[\alpha]^{20}_D$ = - 4.3 (MeOH).

(–)-Pinanediol (1*S*)-1-[3-(methoxycarbonyl)phenylmethanesulfonylamino]-propaneboronate (**5d**). Yellowish solid (56%). ^{1}H NMR (400 MHz, $CDCl_3$) δ: 8.08 (s, 1H, CH_{phen}), 8.04 (dt, *J* = 7.8, 1.5 Hz, 1H, CH_{phen}), 7.64 (dt, *J* = 7.7, 1.5 Hz, 1H, CH_{phen}), 7.45 (t, *J* = 7.7 Hz, 1H, CH_{phen}), 4.35 (dd, *J* = 8.8, 2.1 Hz, 1H, CH_{pin}), 4.32 (d, *J* = 3.3 Hz, 2H, CH_2SO_2), 4.27 (d, *J* = 6.4 Hz, 1H, NH), 3.90 (s, 3H, OCH_3), 3.11 (q, *J* = 5.9 Hz, 1H, CHB), 2.42 (m, 1H, $CH_{2\text{-}pin}$), 2.18 (m, 1H, $CH_{2\text{-}pin}$), 2.06 (t, *J* = 5.5 Hz, 1H, CH_{pin}), 1.93 (tt, *J* = 5.7, 3.0 Hz, 1H, CH_{pin}), 1.84 (ddd, *J* = 14.7, 3.4, 2.1 Hz, 1H, $CH_{2\text{-}pin}$), 1.72 (m, 2H, CH_2), 1.41 (s, 3H, $CH_{3\text{-}pin}$), 1.30 (s, 3H, $CH_{3\text{-}pin}$), 1.11 (d, *J* = 11.0 Hz, 1H, $CH_{2\text{-}pin}$), 0.93 (t, *J* = 7.4 Hz, 3H, CH_3), 0.84 (s, 3H, $CH_{3\text{-}pin}$). ^{13}C NMR (101 MHz, $CDCl_3$) δ: 166.68, 135.30, 132.04, 130.81, 130.39, 129.89, 128.88, 87.27, 78.71, 59.08, 52.36, 51.25, 39.58, 38.36, 35.41, 28.74, 27.16, 26.59, 25.82, 24.14, 10.93. ^{11}B NMR (400 MHz, $CDCl_3$) δ: 32.46. $[\alpha]^{20}_D$ = - 6.7 (MeOH).

(+)-Pinanediol (1*R*)-1-[3-(methoxycarbonyl)phenylmethanesulfonylamino]-propaneboronate (**5e**). Yellowish solid (61%). Spectroscopic properties were identical to **5d**. $[\alpha]^{20}_D$ = + 6.4 (MeOH).

General procedure for the deprotection of boronic esters **5a** and **5c**. Sulfonamidoboronates **5** (0.36 mmol, 1 eq.) were dissolved in dry CH_3CN (2 mL). HCl 3M (0.54 mmol, 1.5 eq.) and isobutylboronic acid (0.34 mmol, 0.95 eq.) were added. The mixture was stirred for 5 min, then *n*-hexane (2 mL) was added. The biphasic mixture was vigorously stirred and after 30 min the *n*-hexane layer, containing the pinanediol isobutylboronate, was removed and fresh *n*-hexane (2 mL) was added. The last procedure was repeated several times until the disappearance of isobutylboronate from the *n*-hexane layer, monitored by TLC analysis (total reaction time 3-6 hours). The acetonitrile phase was then concentrated and the crude recrystallized to afford the sulfonamidoboronic acid.

(1*S*)-1-(Phenylmethanesulfonylamino)-ethaneboronic acid (**6a**). White solid (75%). ^{1}H NMR (400 MHz, MeOD) δ: 7.42 – 7.33 (m, 5H, CH_{phen}), 4.39 – 4.23 (m, 2H, CH_2SO_2), 2.86 (d, *J* = 7.6 Hz, 1H, CHB), 1.14 (d, *J* = 7.4 Hz, 3H, CH_3). ^{13}C NMR (101 MHz, MeOD) δ: 132.38, 132.05, 129.94, 129.79, 62.06, 60.51 (CB), 18.80.^{11}B NMR (400 MHz, $CDCl_3$) δ: 28.82. HRMS $[M]^-$ calc. for $C_9H_{14}BNO_4S$ 243.0737, found 242.0662. $[\alpha]^{20}_D$ = - 20.0 (MeOH).

(1*S*)-1-(Phenylmethanesulfonylamino)-propaneboronic acid (**6c**). White solid (66%). ^{1}H NMR (400 MHz, MeOD) δ: 7.39 (dq, *J* = 17.9, 3.4, 2.9 Hz, 5H, CH_{phen}), 4.38 – 4.22 (m, 2H, CH_2SO_2), 2.79 (t, *J* = 6.6 Hz, 1H, CHB), 1.53 (ddq, *J* = 27.8, 13.8, 7.0 Hz, 2H, CH_2), 0.87 (t, *J* = 7.4 Hz, 3H, CH_3). ^{13}C NMR (101 MHz, MeOD) δ: 131.98, 131.63, 129.54, 129.38, 60.08, 44.28 (CB), 26.34, 11.29. ^{11}B NMR (400 MHz, MeOD) δ: 29.06. HRMS $[M]^-$ calc. for $C_{10}H_{16}BNO_4S$ 257.0893, found 256.0821. $[\alpha]^{20}_D$ = -22.9 (MeOH).

General procedure for deprotection of carboxylic esters **5b**, **5d** and **5e**

Degassed HCl 3M (3.6 mL) was used to dissolve the sulfonamidoboronates **5** (0.33 mmol, 1 eq.). The solution was refluxed for 2-3h. After cooling, the reaction mixture was diluted with water (2 mL) and washed three times with Et_2O (3 × 5 mL). The organic phase was washed twice with water. All the aqueous phase was concentrated in vacuo and CH_2Cl_2 was used to eliminate all the water, affording the desired final product **6**.

(1*S*)-1-[(3-Carboxyphenyl)methanesulfonylamino)-ethaneboronic acid (**6b**). White solid (85%). ^{1}H NMR (400 MHz, MeOD) δ: 8.08 (st, *J* = 1.8 Hz, CH_{phen}), 8.02 (dt, *J* = 7.8, 1.5 Hz, CH_{phen}), 7.66 (dt, *J* = 7.8, 1.5 Hz, CH_{phen}), 7.49 (t, *J* = 7.7 Hz, 1H, CH_{phen}), 4.39 - 4.35 (m, 2H, CH_2SO_2), 2.86 (d, *J* = 7.6 Hz, 1H, CHB), 1.14 (d, *J* = 7.4 Hz, 3H, CH_3). ^{13}C NMR (400 MHz, MeOD) δ: 169.23, 136.46, 133.20, 132.34, 132.32, 130.59, 129.89, 59.65, 38.28 (CB), 18.41. HRMS $[M]^-$ calc. for $C_{10}H_{14}BNO_6S$ 287.0635, found 286.0564. $[\alpha]^{20}_D$ = - 25.2 (MeOH).

(1*S*)-1-[(3-Carboxyphenyl)methanesulfonylamino)-propaneboronic acid (**6d**). White solid (42%). ^{1}H NMR (400 MHz, DMSO-d_6) δ: 12.97 (s, 1H, COOH), 7.97 (d, *J* = 1.8 Hz, 1H, CH_{phen}), 7.90 (dt, *J* = 7.7, 1.5 Hz, 1H, CH_{phen}), 7.81 (s, 2H, $B(OH)_2$), 7.59 (dt, *J* = 7.7, 1.5 Hz, 1H, CH_{phen}), 7.48 (t, *J* = 7.7 Hz, 1H, CH_{phen}), 6.23 (d, *J* = 6.9 Hz, 1H, NH), 4.38 (s, 2H, CH_2SO_2), 2.78 (q, *J* = 6.3 Hz, 1H, CHB), 1.51 (dt, *J* = 9.7, 6.9 Hz, 2H, CH_2), 0.80 (t, *J* = 7.4 Hz, 3H, CH_3). ^{13}C NMR (101 MHz, DMSO-d_6) δ: 166.84, 134.92, 131.47, 131.03, 130.56, 128.50, 128.24, 57.13, 44.55, 24.92, 10.69. ^{11}B NMR (400 MHz, DMSO-d_6) δ: 32.90. HRMS $[M]^-$ calc. for $C_{11}H_{16}BNO_6S$ 301.0791, found 300.0720. $[\alpha]^{20}_D$ = - 27.6 (MeOH).

(1*R*)-1-[(3-Carboxyphenyl)methanesulfonylamino)-propaneboronic acid (**6e**). White solid (52%). Spectroscopic properties were identical to **6d**. HRMS $[M]^-$ calc. for $C_{11}H_{16}BNO_6S$ 301.0791, found 300.0722. $[\alpha]^{20}_D$ = + 27.1.

Supplemental Figure S1. Stereoviews of OXA-24/40 and ADC-33 in complex with BATSIs. Polder omit electron density maps (contoured at 5.5 σ) are drawn as gray cages surrounding the BATSI inhibitors.

A. OXA-24/40 in complex with **CR167**

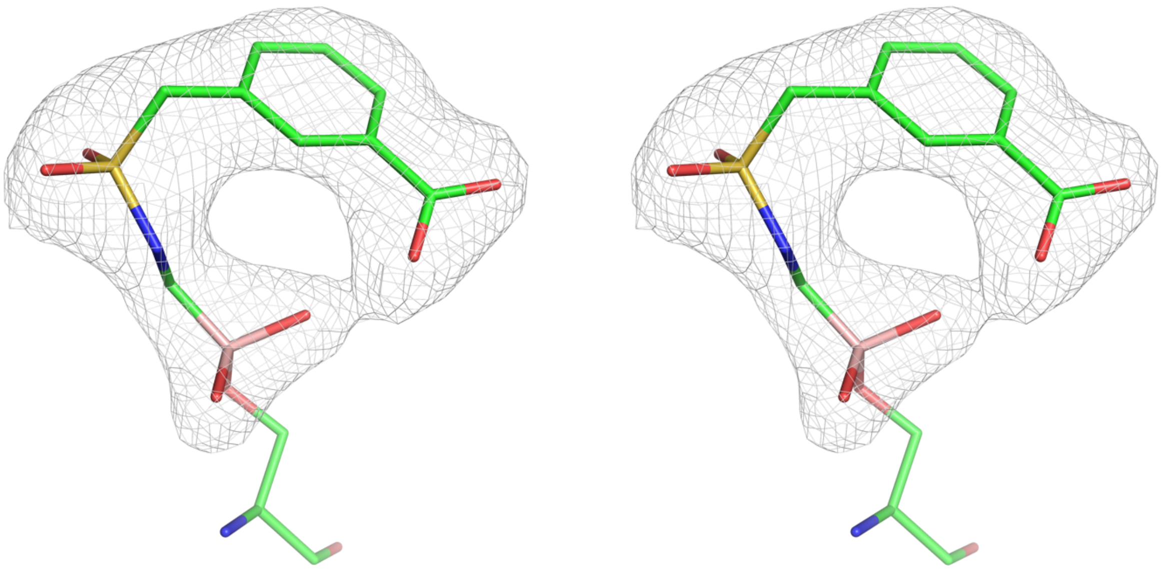

B. ADC-33 in complex with **6d**

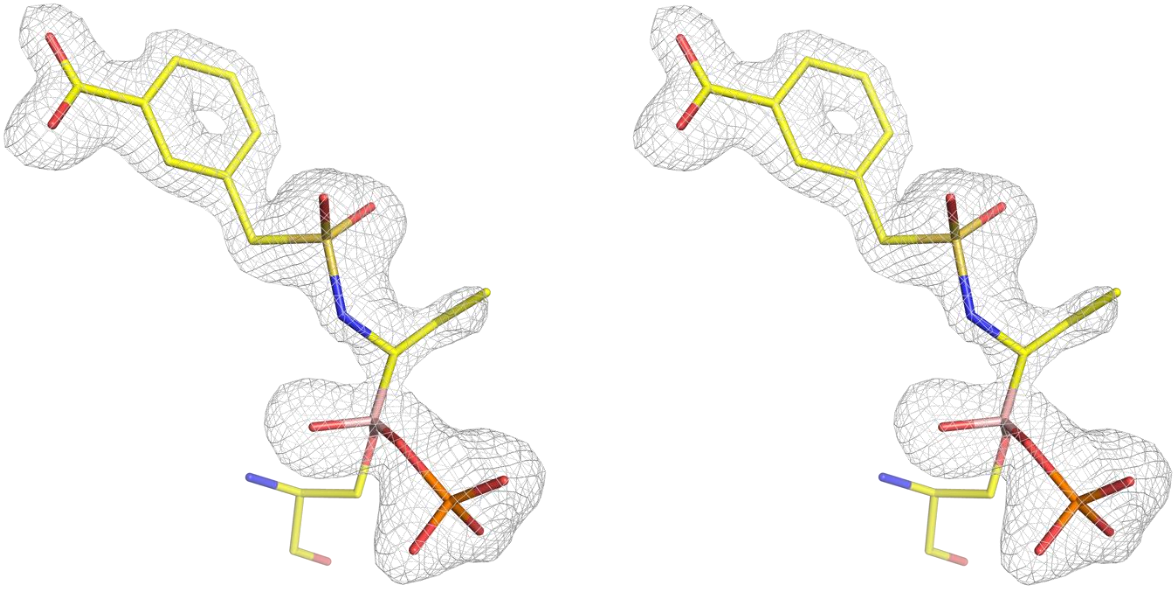

C. ADC-33 in complex with **6e**

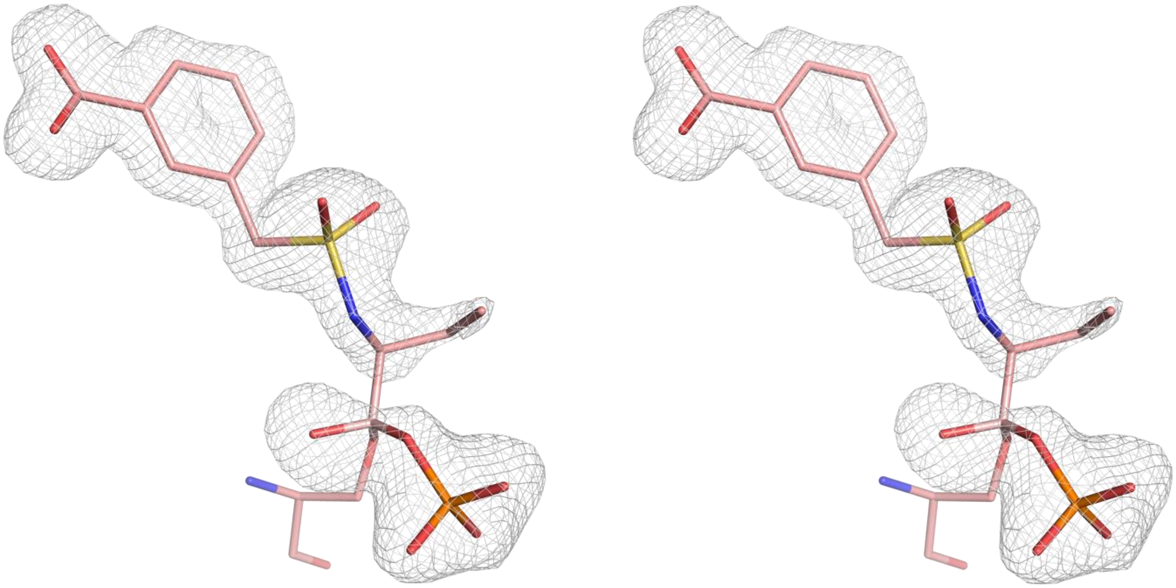

D. OXA-24/40 in complex with **6d**

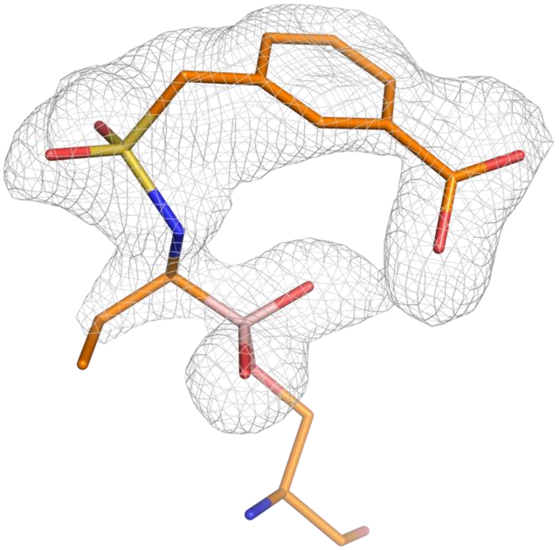

E. OXA-24/40 in complex with **6e**

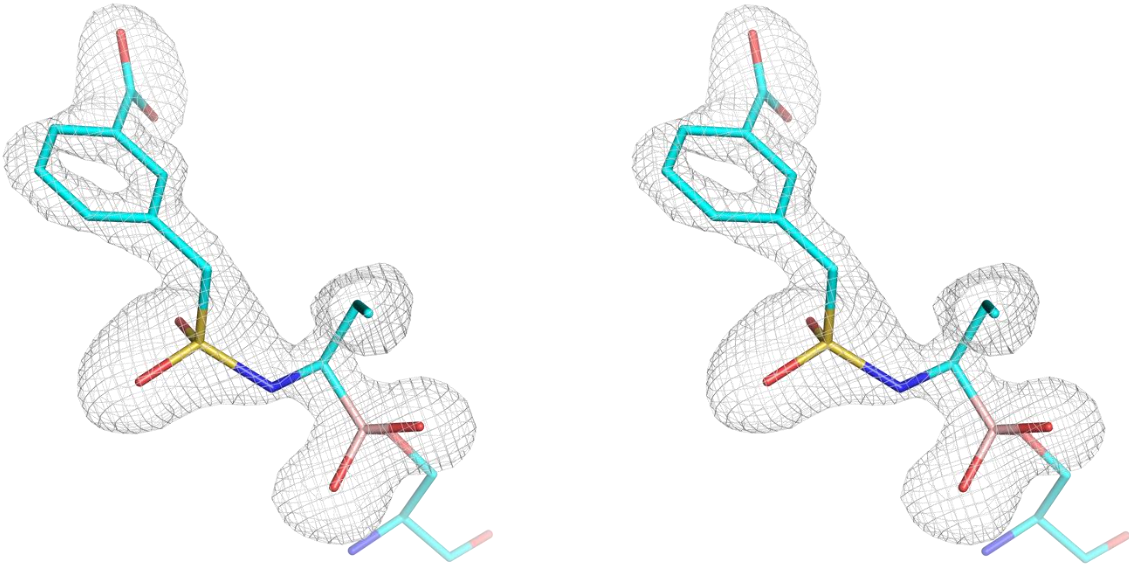

Supplement: Supplementary file 1 [file antibiotics-12-00644-s001.zip › antibiotics-2283409-supplementary.pdf]
